# Continuous Spinal Anesthesia for Labor Analgesia and Cesarean Delivery in a Parturient With Familial Dilated Cardiomyopathy: A Case Report

**DOI:** 10.7759/cureus.48877

**Published:** 2023-11-16

**Authors:** Michiko Sugita, Kazuko Shimizu, Naoyuki Hirata

**Affiliations:** 1 Department of Anesthesiology, Kumamoto University Hospital, Kumamoto, JPN

**Keywords:** ppcm, continuous spinal anesthesia, cesarean delivery, labor analgesia, familial dilated cardiomyopathy

## Abstract

We report a case of successful continuous spinal anesthesia (CSA) for labor analgesia and cesarean delivery in a patient with familial dilated cardiomyopathy (DCM). A 33-year-old pregnant woman diagnosed with DCM was scheduled for a vaginal delivery under labor analgesia. An accidental intrathecal catheter was placed, and labor analgesia was provided by CSA. The vaginal delivery was converted to a cesarean delivery, and an intrathecal catheter was used for transition, which avoided hemodynamic changes and allowed the patient to safely undergo cesarean delivery. CSA is a reliable and rapidly titratable technique that provides excellent analgesia without hemodynamic changes in patients with DCM undergoing labor analgesia and subsequent cesarean delivery.

## Introduction

Anesthetic management of labor and delivery in patient with dilated cardiomyopathy (DCM) is not well defined. The choice of anesthetic technique in these patients depends on the clinical status and the ability to maintain hemodynamic goals during the peripartum period [1].

Analgesia for labor during vaginal delivery can be useful to avoid changes in the circulatory dynamics of patients with cardiac complication. A transition from vaginal delivery to cesarean delivery is often required depending on the mother and infant situation. On the other hand, spinal anesthesia is generally used for labor analgesia and cesarean delivery and provides adequate analgesia. However, it is difficult to adjust the dosage of anesthetics to avoid circulatory changes, especially in pregnant women with cardiac complications. Continuous spinal anesthesia (CSA) can afford excellent anesthesia with minimal hemodynamic perturbation and can be accomplished by a very slow titration of local anesthetics allowing gradual patient compensation to the physiologic changes of a sympathetic blockade [2]. In this report, we describe a case in which CSA was safely performed in a pregnant woman with familial DCM, both for labor analgesia and for the transition to cesarean delivery.

## Case presentation

**Figure 1:**
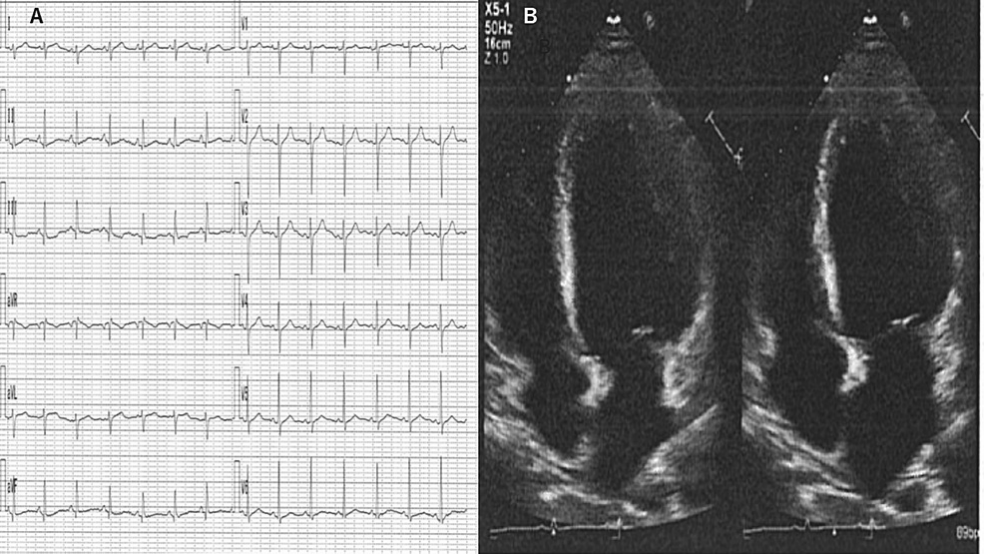
Electrocardiogram and echocardiography A: Electrocardiogram at 35 weeks' gestation showed sinus rhythm and T-wave abnormalities. B: Echocardiography at 35 weeks' gestation showed left ventricular enlargement, decreased left ventricular wall motion, and  pericardial effusion.

A 33-year-old pregnant woman (gravida 2, para 0, height 164 cm, and weight 78 kg) who was diagnosed with DCM four years ago consulted to the anesthesia department for perinatal management. She had a family history of DCM; her father had recurrent cardiac failure symptoms, and her sister suspected peripartum cardiomyopathy (PPCM) and still limited physical exercise. The patient's cardiac function was determined as a left ventricular ejection fraction (LVEF) of 29.3% at 35 weeks of gestation and the New York Heart Association functional classification of class I under no medication (Figure 1). The pregnancy course was uneventful.

Vaginal delivery under labor analgesia and elective induction of labor was planned at 38 weeks’ gestation. Her cardiac risk at delivery was ventricular arrhythmia associated with elevated catecholamine levels. In addition to standard monitoring, a defibrillator was kept on standby, and a pad was attached in case of severe ventricular arrhythmia. Labor analgesia procedure was performed when the cervix was 4 cm dilated. After confirming the epidural space using the loss of resistance method with an 18-gauge Tuohy needle, the dura mater was punctured with a 27-gauge pencil point needle for dual puncture epidural methods. A porosity catheter was then smoothly inserted via the Tuohy needle. As a test dose, 2 mL of 1% lidocaine was administered, and the patient immediately complained of an abnormal sensation in her hip. We suspected that the catheter strayed into the subarachnoid space, and a suction test confirmed persistent fluid reflux. Analgesia below Th10 and motor blockade at Bromage scale 3 were observed. The catheter tip was determined to be in the intrathecal space. We considered removal of the catheter and re-puncture. However, in this case, we chose to employ CSA for the following three reasons. First, sufficient analgesia was needed to suppress the catecholamine levels during delivery. Second, instrumented delivery was planned to avoid pushing, so motor block was acceptable. Third, it would be easier to adjust the anesthetic level for conversion to cesarean anesthesia. In addition, because drug misadministration is likely to cause serious side effects, the drugs were administered by an anesthesiologist until the end of delivery.

Labor pain disappeared after administration of a test dose of lidocaine. Isobaric bupivacaine (0.1%) and fentanyl (5 µg/mL) were continuously administered at 1.3-2.0 mL/h into the intrathecal space 80 min after lidocaine test dose administration. After the start of CSA, the numerical rating scale remained at 0-2, and Bromage scale was 2-3. No major circulatory changes and echocardiogram examination were observed. One hour after the start of labor analgesia, transient fetal bradycardia was observed, and the decision was made to perform a cesarean delivery three hours 30 minutes later. The level of anesthesia was in the Th10-S.

Twenty minutes after the decision to perform cesarean delivery, the patient was transferred to the operating room. After the intravenous administration of famotidine 20 mg and metoclopramide 10 mg, an arterial catheter was placed to measure arterial pressure, and arterial pressure-based cardiac output was monitored (FloTrac SensorTM; Edwards Lifesciences Corporation, Irvine, CA, USA). Doses of 2.5 mg of 0.5% hyperbaric bupivacaine, 15 µg of fentanyl, and 150 µg of morphine were administered via an intrathecal catheter. Five minutes later, as the anesthetic level of Th10 remained, bupivacaine 2.5 mg was additionally administered. Continuous phenylephrine infusion was started at 0.2 µg/kg/min at the same time as anesthetic administration. The hemodynamic status remained stable (Table 1). Twelve minutes after the initial anesthetic administration, analgesia level was below Th6. Before delivery, 100 µg of nitroglycerin was administered to reduce auto-transfusion after delivery. A baby was delivered 12 min after the start of surgery. The Apgar scores were 8 and 9 at one and five minutes, respectively, and the pH of the umbilical artery was 7.284. Intravenous oxytocin was administered continuously at a rate of 10 units/h. The hemodynamic status remained stable after delivery. The operation time was 71 min, the infusion volume of crystalloids was 900 mL, and the blood loss, including amniotic fluid, was 555 mL. The intrathecal catheter was removed after the surgery.

**Table 1:** Hemodynamic measurements during labor HR: heart rate, BP: blood pressure, S/D: systolic/diastolic, CO: cardiac output, CI: cardiac index

	Baseline	Labor analgesia	Cesarean delivery	ICU
HR (beats/min)	82	75-100	99-121	78-86
BP S/D (mmHg)	126/77	105/148/60-80	107-132/64-75	119-149/62-80
CO (L/min)	-	-	6.0-7.8	4.4-7.9
CI (L/min/m^2^)	-	-	3.2-4.2	3.0-4.3

Postoperatively, the patient was managed in the intensive care unit (ICU). No circulatory changes or arrhythmias were observed after admission to the ICU. Postoperative analgesia was well controlled with intrathecal morphine and intravenous acetaminophen. Moreover, there were no symptoms of post-dural puncture headache or meningitis. Her cardiac function tended to recover after delivery (Table 2). After delivery, cabergoline, bisoprolol, and valsartan were administered to prevent cardiac exacerbation. The patient was discharged on the seventh day after delivery. There was no deterioration of cardiac function at the three-month follow-up.

**Table 2:** Echocardiographic findings and BNP MR: mitral regurgitation, BNP: brain natriuretic peptide, LVEF: left ventricular ejection fraction

Time	LVEF (%)	Other findings	BNP (pg/mL)
Before pregnant	48.8	Left ventricular enlargement	23.1
12w	44.3	Left ventricular enlargement	29.3
30w	37.1	Left ventricular enlargement	18.4
35w	29.3	Left ventricular enlargement, pericardial effusion	28.0
3 days after delivery	42.1	Mild MR, left atrial enlargement, pleural effusion	97.7
3 months after delivery	40.3	Mild MR, no pleural effusion	65.3

 Written informed consent was obtained from the patient for the publication of this case report.

## Discussion

DCM is characterized by an enlarged left ventricle and systolic dysfunction. Idiopathic DCM accounts for approximately 50% of all cases, and 35% are hereditary [3]. It is recommended that pregnancy should be discouraged if LVEF is less than 30%; however, patients with mild left ventricular dysfunction and good functional class can tolerate pregnancy [4]. Ituk et al. reported 24 deliveries complicated with DCM. Eight were delivered vaginally under labor epidural analgesia, and one was a sudden accidental vaginal delivery. Seven cesarean deliveries were performed under combined spinal-epidural anesthesia, five were performed under epidural anesthesia, including three that got converted from vaginal delivery, and three patients had general anesthesia [1].

In our case, the patient was diagnosed with familial DCM before pregnancy, and cardiac dysfunction was observed. Therefore, it was important to avoid hemodynamic change and elevation of catecholamine level during delivery. Neuraxial analgesia blocks the sympathetic nervous system, thus decreasing vascular resistance and causing hypotension. To avoid hemodynamic change, titration of anesthetic effects would be ideal. In this regard, CSA can be titrated and provides excellent analgesia under minimal hemodynamic effects. CSA has been reported to be useful in the delivery of pregnant women complicated with cardiac disease [5,6,7]. In our case, low-concentration bupivacaine and fentanyl were administered intrathecally for labor analgesia with cardiovascular stability, and the transition from labor analgesia to a cesarean delivery was performed by CSA successfully. The disadvantages of CSA include post-dural puncture headache, infection, and incorrect drug administration. In this case, because a dural puncture was not performed by a Tuohy needle but a catheter, little or no leakage of spinal fluid existed presumably.

Fortunately, the patient did not suffer from post-dural puncture headaches. No cases of infections due to intrathecal catheters have been reported in obstetric meta-analyses or other studies [8,9]. In the present case, only prophylactic antibiotics were administered, and no signs of meningitis were observed. If the drug is mistakenly administered through a catheter, a total spinal block can occur, which can be serious in high-risk cases. Labeling is important for the misidentification and misadministration of catheters [10]. In this case, to avoid misadministration, we limited drug administration to only one anesthesiologist.

## Conclusions

CSA is a reliable and rapid titration technique and provides excellent analgesia with minimal undesirable hemodynamic changes. The present case demonstrates that CSA can be successfully employed for patient with DCM undergoing labor analgesia and transitioning to cesarean delivery. CSA should be considered an anesthetic option for labor and delivery of pregnant women with cardiac diseases.
